# Supplementary material for: ATAC-seq with unique molecular identifiers improves quantification and footprinting
Source: Commun Biol. 2020 Nov 13;3:675. doi: 10.1038/s42003-020-01403-4 (PMC7666144; doi:10.1038/s42003-020-01403-4)
Supplement: Supplementary file 1 — Supplementary Information [file 42003_2020_1403_MOESM1_ESM.pdf]

Supplementary information

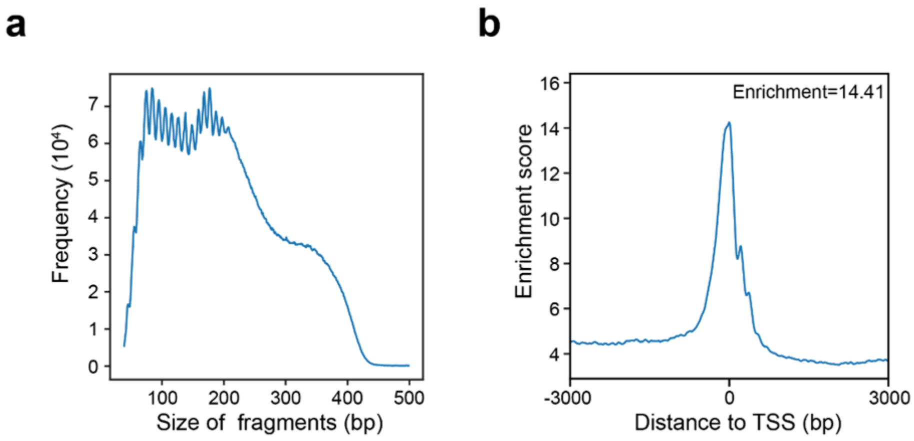

Supplementary Figure 1: The quality control results of UMI-ATAC-seq

(a) Distribution of fragment sizes for sample C019 from aligned, filtered and deduplicated reads. (b) Enrichment of ATAC-seq accessibility near Transcription Start Sites (TSS) in sample C019.

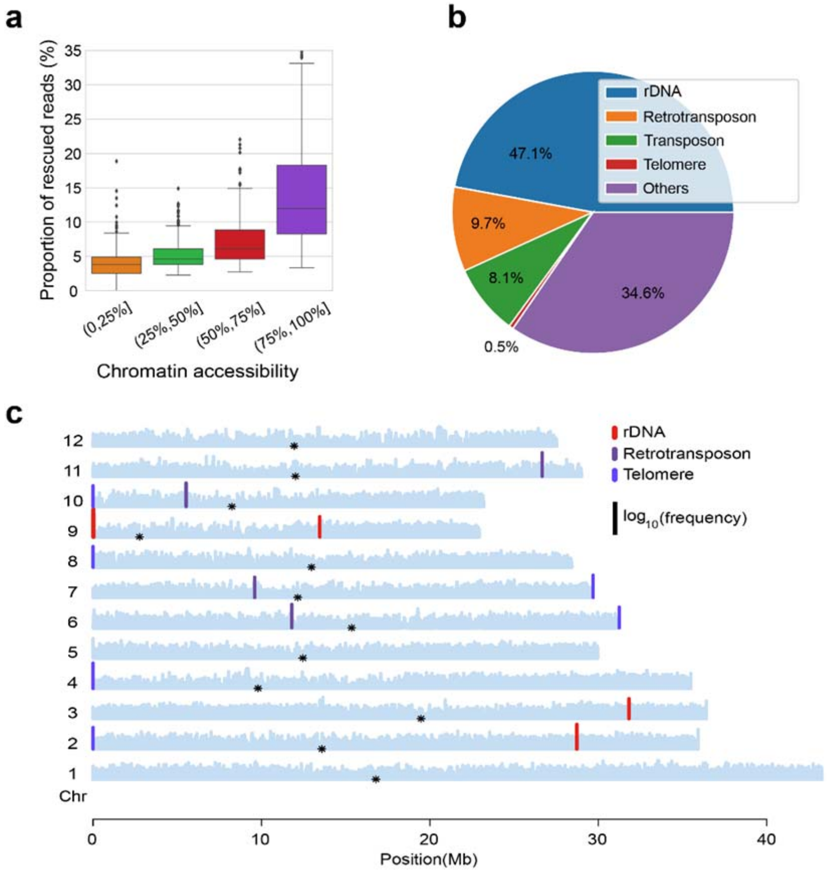

Supplementary Figure 2: The characteristics of rescued reads which have identical mapping coordinates but different UMI in sample C019.

(a) Box plot illustrating the distribution of rescued reads with different chromatin accessibility. We divided the genome into 1 kb bins and counted the rescued reads in each bin. Then we grouped all bins by 25%, 50% and 75% quantiles. Only reads with mapping quality greater than thirty were used. (b) The annotations of rescued reads. (c) The distribution of rescued reads in the genome. The maximum value of the frequency in a bin (1 kb) is 39,912. Bins annotated as rDNA, retrotransposon and telomere are marked. Black points represent the

centromere.

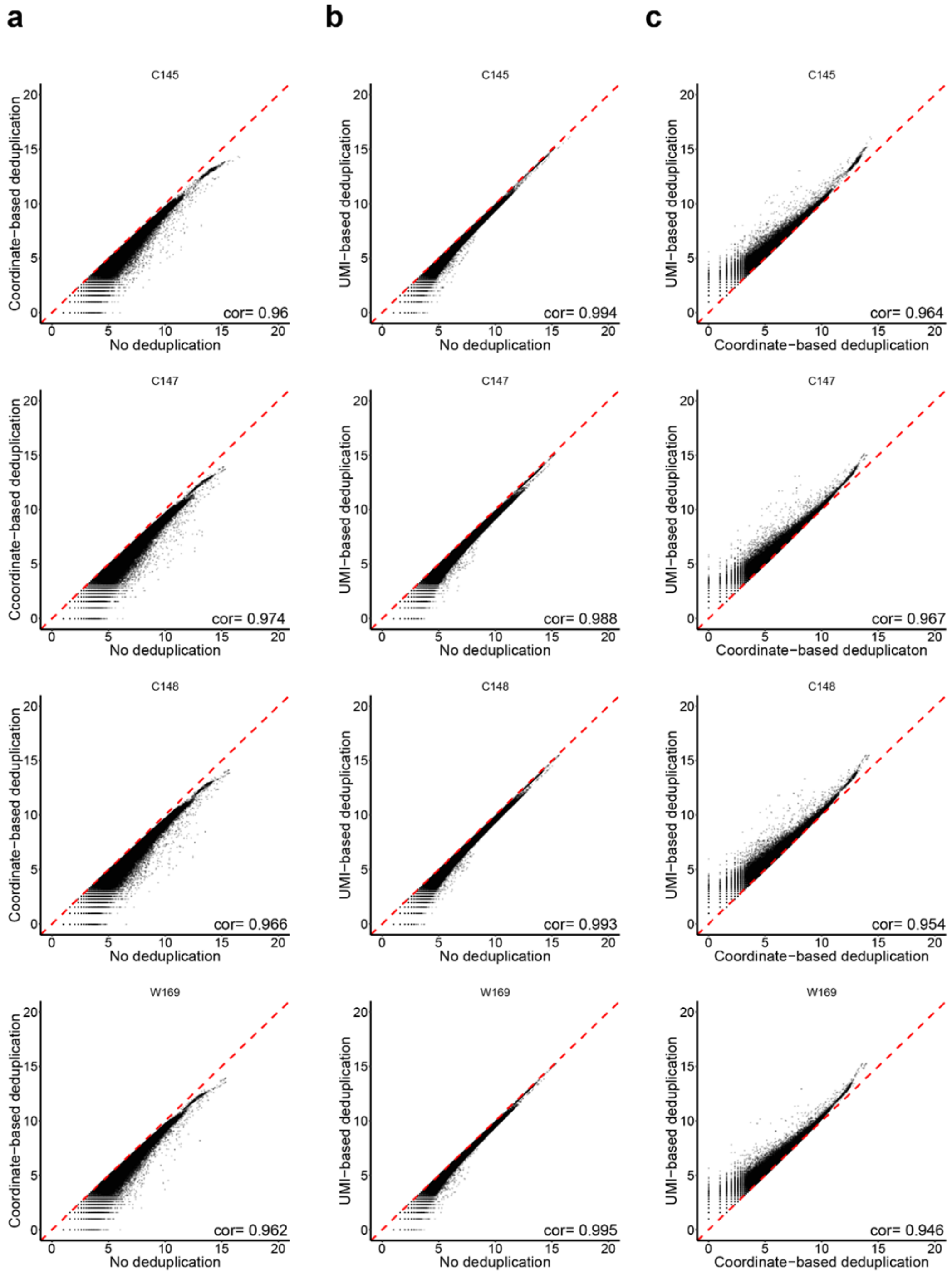
a
b
c
C145
C147
C148
W169
Coordinate−based deduplication
Ccoordinate−based deduplication
UMI−based deduplication
No deduplication
Coordinate−based deduplication
Coordinate−based deduplicaton
cor= 0.96
cor= 0.994
cor= 0.964
cor= 0.974
cor= 0.988
cor= 0.967
cor= 0.966
cor= 0.993
cor= 0.954
cor= 0.962
cor= 0.995
cor= 0.946
0
5
10
15
20

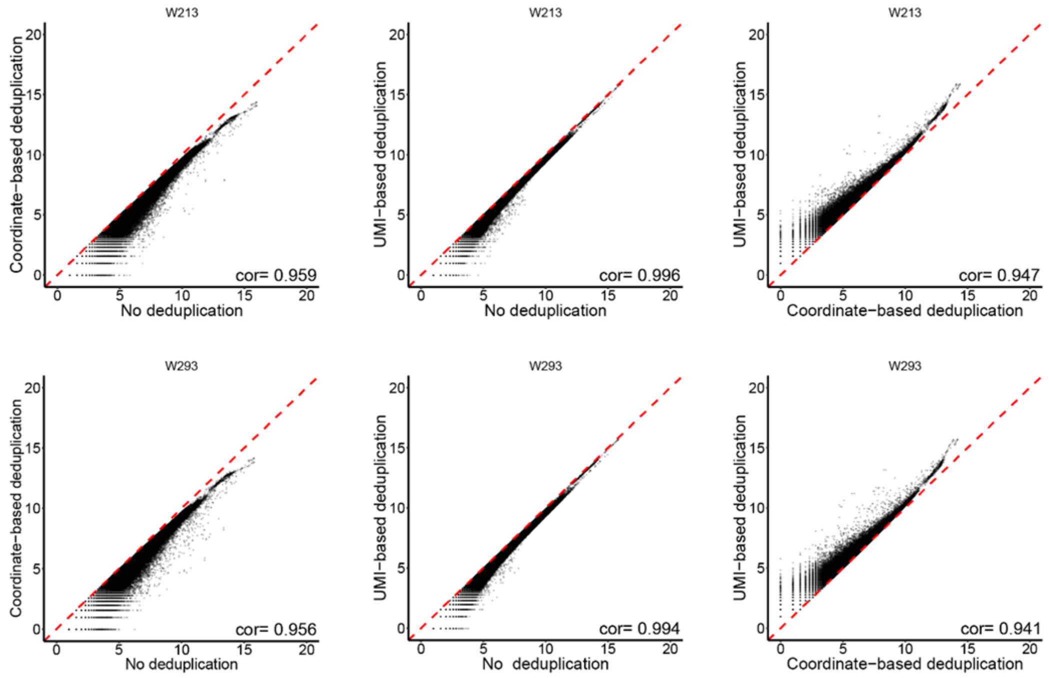

Supplementary Figure 3: The correlations of quantification by removing PCR duplicates in different ways in different samples

Each point is the number of Tn5 insertions in a 250 bp bin (log base 2). (a) Scatter plots contrasting quantification results by mapping coordinate-based deduplication (CD) and no deduplication (ND). (b) Scatter plots contrasting quantification result by UMI-based deduplication (UD) and ND. (c) Scatter plots contrasting quantification result by UD and CD.

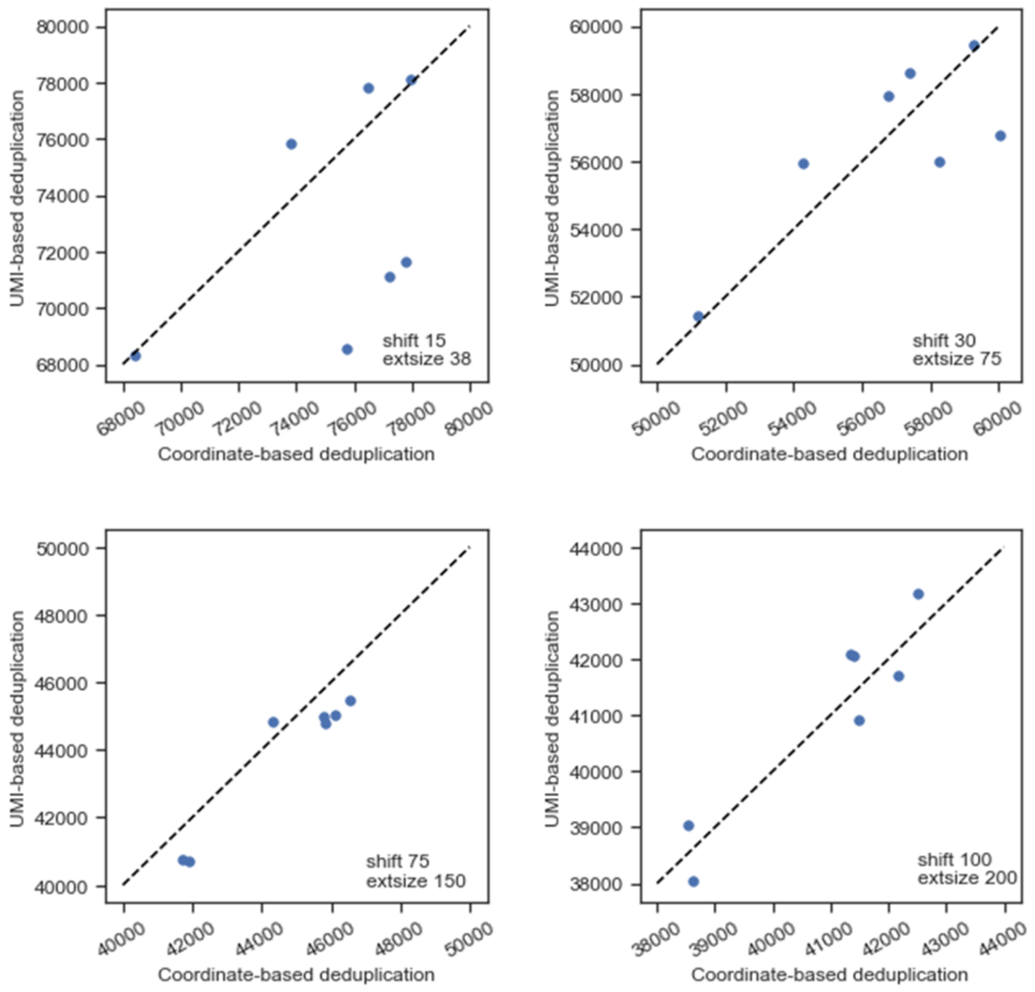

Supplementary Figure 4: The comparisons of peak number generated by removing PCR duplicates in different ways in different samples.

The parameters of calling peaks are showed in the lower right corner of each diagram.

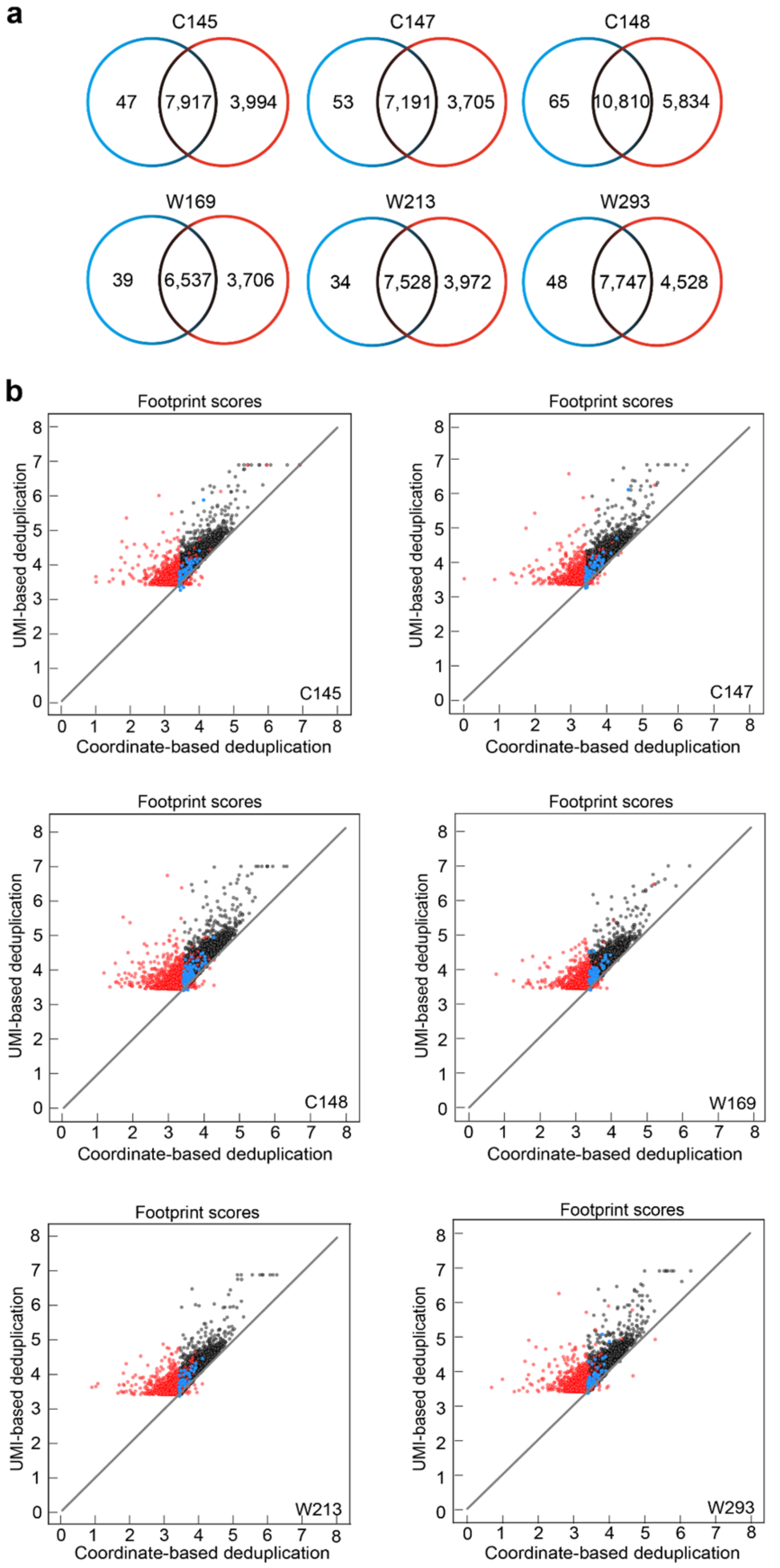
a
C145
47
7,917
3,994
C147
53
7,191
3,705
C148
65
10,810
5,834
W169
39
6,537
3,706
W213
34
7,528
3,972
W293
48
7,747
4,528
b
Footprint scores
UMI-based deduplication
Coordinate-based deduplication
C145
C147
C148
W169
W213
W293

Supplementary Figure 5: The comparisons of footprints identified by removing PCR duplicates in different ways in different samples

(a) Venn diagram displaying the footprints identified by pyDNase (Wellington Footprint *P-value* < 10^{-30}) with CD (blue) and UD (red). The common footprints (black) are defined as those overlapped by at least one base. (b) The relationship between footprint scores calculated based on CD and UD in different samples. The footprint scores are calculated as $log(-log_{10}(P\text{-}value))$. The colors of points are the same as the Venn diagram in (a). The sample names are showed in the lower right corner of each diagram.

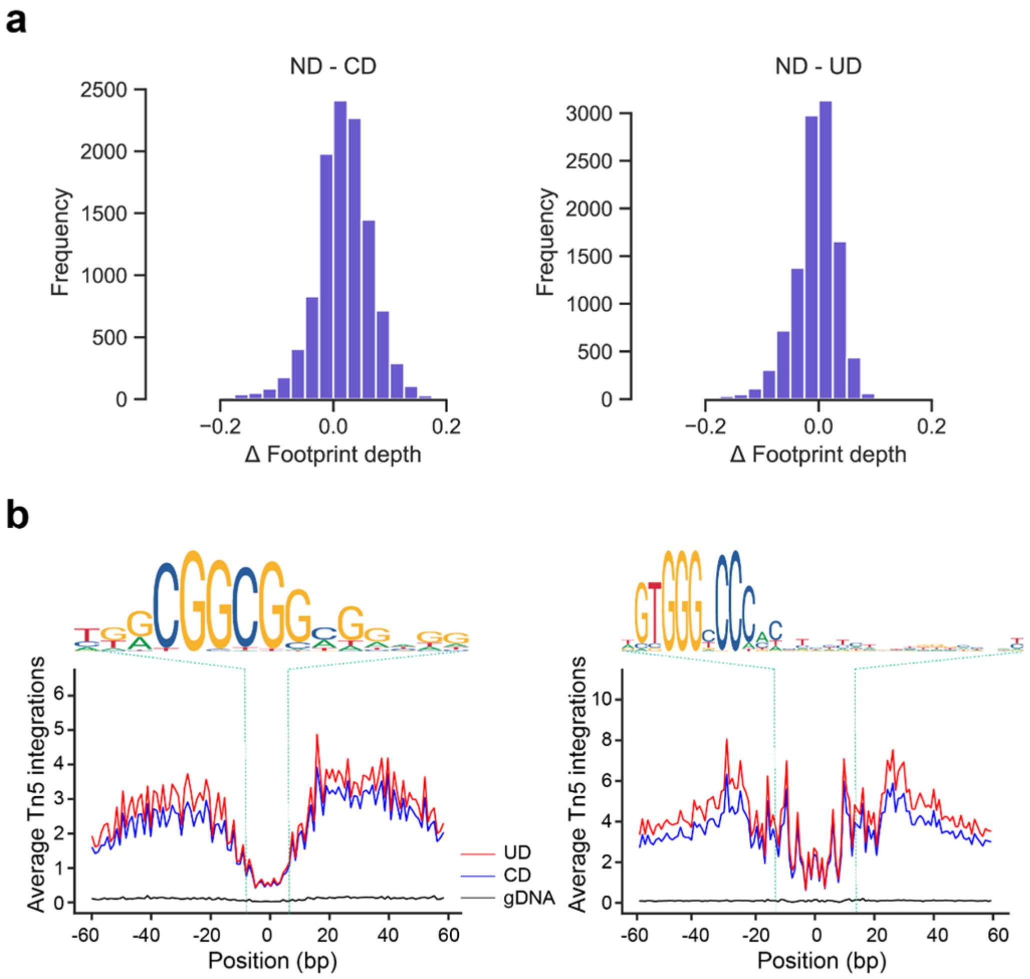

Supplementary Figure 6: The comparisons of footprint pattern with removing PCR duplicates in different ways in sample C109.

(a) The distribution of the differences in FPD values between ND and CD or ND and UD datasets. (b) Averaged Tn5 insertions per base for AP2/ERF (left) and TCP1(right) motif sites, which are overlapped with common pyDNase footprints identified in both CD and UD modes and located in the top 25% of the most accessible regions. The motifs are shown at the top of each diagram. Vertical lines (green) depict the edges of the motif match. The black line is genomic DNA sequencing data of

Tn5-tagmented libraries (14.6-fold coverage).

Supplementary Table 1: Primers used for UMI-ATAC-seq

Application	Primer name	Primer sequence
Tn5 assembly (ME sequence denotes in green; UMI sequence denotes in red; N denotes a random base)	MErev	[phos]CTGTCTCTTATACACATCT
	ME-A6	CGACGCTCTTCCGATCTNNNNNNAGATGTGTATAAGAGACAG
	ME-A13	CGACGCTCTTCCGATCTNNNNNNNNNNNNNAGATGTGTATAAGAGACAG
	ME-A20	CGACGCTCTTCCGATCTNNNNNNNNNNNNNNNNNNNNAGATGTGTATAAGAGACAG
	ME-A25	CGACGCTCTTCCGATCTNNNNNNNNNNNNNNNNNNNNCTGCTAGATGTGTATAAGAGACAG
	ME-B	GTCTCGTGGGCTCGGAGATGTGTATAAGAGACAG
PCR	PCR Primer 1	AATGATACGGCGACCACCGAGATCTACACACACTCTTTCCCTACACGACGCTCTTCCGA
	Barcoded PCR Primer 2 (barcodes denote in red)	CAAGCAGAAGACGGCATACGAGATTGCCTCTTGTCTCGTGGGCTCGGAGATGT
		CAAGCAGAAGACGGCATACGAGATTCCTCTACGTCTCGTGGGCTCGGAGATGT
		CAAGCAGAAGACGGCATACGAGATATCACGACGTCTCGTGGGCTCGGAGATGT
		CAAGCAGAAGACGGCATACGAGATACAGTGGTGTCTCGTGGGCTCGGAGATGT
		CAAGCAGAAGACGGCATACGAGATCAGATCCAGTCTCGTGGGCTCGGAGATGT
		CAAGCAGAAGACGGCATACGAGATACAAACGGGTCTCGTGGGCTCGGAGATGT
		CAAGCAGAAGACGGCATACGAGATACCCAGCAGTCTCGTGGGCTCGGAGATGT
Sequence	Read1 Sequencing Primer/TrueSeq	ACACTCTTTCCCTACACGACGCTCTTCCGATCT

Supplementary Table 2: The summary statistics for UMI-ATAC-seq data

Sample	Total read pairs	Mapping rate	Coordinate-based duplication rate	UMI-based duplication rate	UMI rescue rate	Fraction of reads in peaks (FRiP)	TSS enrichment
C019	31,959,021	94.89%	33.43%	25.74%	23.00%	65.67%	14.41
C145	31,348,458	94.52%	36.25%	29.36%	19.01%	62.91%	14.04
C147	32,109,151	95.76%	43.74%	38.05%	13.01%	63.91%	13.97
C148	37,431,383	95.37%	37.26%	30.55%	18.01%	63.59%	13.45
W169	24,208,294	95.76%	34.19%	27.69%	19.01%	66.81%	15.25
W213	34,403,390	95.51%	35.78%	28.98%	19.01%	60.88%	13.34
W293	29,594,417	94.24%	37.83%	31.02%	18.00%	65.41%	14.68
