# Supplementary material for: ATAC-seq with unique molecular identifiers improves quantification and footprinting
Source: Commun Biol. 2020 Nov 13;3:675. doi: 10.1038/s42003-020-01403-4 (PMC7666144; doi:10.1038/s42003-020-01403-4)
Supplement: Supplementary file 3 — Reporting Summary [file 42003_2020_1403_MOESM3_ESM.pdf]

nature research

Corresponding author(s): COMMSBIO-20-0399A

Last updated by author(s): Sep 25, 2020

Reporting Summary

Nature Research wishes to improve the reproducibility of the work that we publish. This form provides structure for consistency and transparency in reporting. For further information on Nature Research policies, see our Editorial Policies and the Editorial Policy Checklist.

Statistics

For all statistical analyses, confirm that the following items are present in the figure legend, table legend, main text, or Methods section.

n/a	Confirmed	
☐	☒	The exact sample size (*n*) for each experimental group/condition, given as a discrete number and unit of measurement
☐	☒	A statement on whether measurements were taken from distinct samples or whether the same sample was measured repeatedly
☐	☒	The statistical test(s) used AND whether they are one- or two-sided<br>*Only common tests should be described solely by name; describe more complex techniques in the Methods section.*
☒	☐	A description of all covariates tested
☒	☐	A description of any assumptions or corrections, such as tests of normality and adjustment for multiple comparisons
☒	☐	A full description of the statistical parameters including central tendency (e.g. means) or other basic estimates (e.g. regression coefficient) AND variation (e.g. standard deviation) or associated estimates of uncertainty (e.g. confidence intervals)
☐	☒	For null hypothesis testing, the test statistic (e.g. F, t, r) with confidence intervals, effect sizes, degrees of freedom and P value noted<br>*Give P values as exact values whenever suitable.*
☒	☐	For Bayesian analysis, information on the choice of priors and Markov chain Monte Carlo settings
☒	☐	For hierarchical and complex designs, identification of the appropriate level for tests and full reporting of outcomes
☐	☒	Estimates of effect sizes (e.g. Cohen's *d*, Pearson's *r*), indicating how they were calculated

Our web collection on statistics for biologists contains articles on many of the points above.

Software and code

Policy information about availability of computer code

Data collection	No software was used for data collection.
Data analysis	UMI-ATAC-seq raw reads were first trimmed by Trimmomatic (v.0.36). Trimmed reads were aligned to the Oryza sativa L.ssp.japonica (cv.Nipponbare) reference genome (v.7.0) using BWA-mem. Sorting aligned reads and removing PCR duplicates with mapping coordinates were conducted using SAMtools (v.1.9). The reads rescued by UMI were aligned to the Oryza Repeat Database using blastn (v.2.5.0). Using the "callpeak" function in MACS2 to call peak. Transcription factor footprints were Identified using pyDNase package (available at https://pythonhosted.org/pyDNase/). IGV (Integrative Genomics Viewer) was utilized for Tn5 insertion events visualization. FIMO (v.5.0.5) was used to scan potential binding sites of transcription factors. The position weight matrices (PWMs) were downloaded from JASPAR36 (http://aspar.genereg.net/downloads/). Codes to remove sequencing adapters and PCR duplicates with UMIs can be obtained from the GitHub repository (https://github.com/tzhu-bio/UMI-ATAC-seq).

For manuscripts utilizing custom algorithms or software that are central to the research but not yet described in published literature, software must be made available to editors and reviewers. We strongly encourage code deposition in a community repository (e.g. GitHub). See the Nature Research guidelines for submitting code & software for further information.

Data

Policy information about availability of data

All manuscripts must include a data availability statement. This statement should provide the following information, where applicable:

- Accession codes, unique identifiers, or web links for publicly available datasets
- A list of figures that have associated raw data
- A description of any restrictions on data availability

UMI-ATAC-seq data from rice young panicles are available at the NCBI BioProject (PRJNA602139)

Field-specific reporting

Please select the one below that is the best fit for your research. If you are not sure, read the appropriate sections before making your selection.

☒ Life sciences ☐ Behavioural & social sciences ☐ Ecological, evolutionary & environmental sciences

For a reference copy of the document with all sections, see nature.com/documents/nr-reporting-summary-flat.pdf

Life sciences study design

All studies must disclose on these points even when the disclosure is negative.

Sample size	A total of 7 rice young panicles were analyzed. Each sample was sequenced with about 30M reads by Illumina HiSeq X-ten in PE150 mode.
Data exclusions	No data were excluded from the analyses.
Replication	The conclusions in this study are based on results that could be successfully reproduced.
Randomization	Samplings of plant tissue were performed by randomly selecting plants grown under the same conditions. All samples were allocated randomly into experimental groups.
Blinding	Blinding is not applicable in this study because it does not involve subjects which receive different treatments.

Reporting for specific materials, systems and methods

We require information from authors about some types of materials, experimental systems and methods used in many studies. Here, indicate whether each material, system or method listed is relevant to your study. If you are not sure if a list item applies to your research, read the appropriate section before selecting a response.

Materials & experimental systems

n/a	Involved in the study
☒	☐ Antibodies
☒	☐ Eukaryotic cell lines
☒	☐ Palaeontology and archaeology
☒	☐ Animals and other organisms
☒	☐ Human research participants
☒	☐ Clinical data
☒	☐ Dual use research of concern

Methods

n/a	Involved in the study
☒	☐ ChIP-seq
☒	☐ Flow cytometry
☒	☐ MRI-based neuroimaging
